# Genome sequencing of high-penicillin producing industrial strain of *Penicillium chrysogenum*

**DOI:** 10.1186/1471-2164-15-S1-S11

**Published:** 2014-01-24

**Authors:** Fu-Qiang Wang, Jun Zhong, Ying Zhao, Jingfa Xiao, Jing Liu, Meng Dai, Guizhen Zheng, Li Zhang, Jun Yu, Jiayan Wu, Baoling Duan

**Affiliations:** New Drug Research and Development Center of North China Pharmaceutical Group Corporation, National Engineering Research Center of Microbial Medicine, Hebei Industry Microbial Metabolic Engineering & Technology Research Center, Shijiazhuang, Hebei 050015 China; CAS Key Laboratory of Genome Sciences and Information, Beijing Institute of Genomics, Chinese Academy of Sciences, Beijing, 100101 China; University of Chinese Academy of Sciences, Beijing, 100049 China

## Abstract

**Background:**

Due to the importance of *Penicillium chrysogenum* holding in medicine, the genome of low-penicillin producing laboratorial strain Wisconsin54-1255 had been sequenced and fully annotated. Through classical mutagenesis of Wisconsin54-1255, product titers and productivities of penicillin have dramatically increased, but what underlying genome structural variations is still little known. Therefore, genome sequencing of a high-penicillin producing industrial strain is very meaningful.

**Results:**

To reveal more insights into the genome structural variations of high-penicillin producing strain, we sequenced an industrial strain *P. chrysogenum* NCPC10086. By whole genome comparative analysis, we observed a large number of mutations, insertions and deletions, and structural variations. There are 69 new genes that not exist in the genome sequence of Wisconsin54-1255 and some of them are involved in energy metabolism, nitrogen metabolism and glutathione metabolism. Most importantly, we discovered a 53.7 Kb "new shift fragment" in a seven copies of determinative penicillin biosynthesis cluster in NCPC10086 and the arrangement type of amplified region is unique. Moreover, we presented two large-scale translocations in NCPC10086, containing genes involved energy, nitrogen metabolism and peroxysome pathway. At last, we found some non-synonymous mutations in the genes participating in homogentisate pathway or working as regulators of penicillin biosynthesis.

**Conclusions:**

We provided the first high-quality genome sequence of industrial high-penicillin strain of *P. chrysogenum* and carried out a comparative genome analysis with a low-producing experimental strain. The genomic variations we discovered are related with energy metabolism, nitrogen metabolism and so on. These findings demonstrate the potential information for insights into the high-penicillin yielding mechanism and metabolic engineering in the future.

**Electronic supplementary material:**

The online version of this article (doi:10.1186/1471-2164-15-S1-S11) contains supplementary material, which is available to authorized users.

## Background

Penicillin and β-lactam antibiotic play a significant role in human medical history [1, 2] since Fleming's discovery of the filamentous fungus *Penicillium notatum* in 1929 [3]. The regulation of penicillin biosynthesis has been studied for many years, together with much more proteins or pathways were discovered [4–9]. The improvement of *P. chrysogenum* strains to obtain higher penicillin yields is a main intense objective in industrial research [10, 11].

Due to the importance of *P. chrysogenum*, the genome sequence of low-penicillin producer Wisconsin54-1255, which is widely used in laboratories, was sequenced and a number of genes responsible for key steps in penicillin production were identified [12]. The precursors for penicillin biosynthesis, genes encoding microbody proteins and transporters were found, illustrating potential for future genomics-driven metabolic engineering [12]. Through classical mutagenesis and screening methods, product titers and productivities of penicillin have dramatically increased since Wisconsin54-1255 strain, but how low-penicillin producer strain was transformed into an efficient producer through improvement is still challenging. For commercial reasons, the improvement of *P. chrysogenum* strains has never been stopped. The productivity of industrial used strains is far more higher than their ancestor, and the progress was mainly obtained by classical mutagenesis and screening methods. Because mutations were random, most of the genetic changes in high yield strains were unclear. Although some significant structural variations (SVs) [8, 9, 13] and differential expression profiling [12, 14, 15] have been found in high-penicillin producing strains, little is known about the underlying whole genomic changes between low-producing laboratorial strain and high-producing industrial strain.

To gain more insight into the genome structural variations of high-penicillin producing strain, we sequenced a Chinese industrial strain NCPC10086. We also offer a comprehensive comparative genomics analysis [16–19] to find all mutations and large-scale structural variations between NCPC10086 and the first published genome of *P. chrysogenum* strain, Wisconsin54-1255 [12]. Some variations including mutations, indels and structural variations were considered for their potential biological impact for penicillin biosynthesis. Our genome sequence data and analyses explore the differences between high- and low-yield *P. chrysogenum* strains and demonstrate the potential useful information to improve strains by direct genetic engineering tools.

## Results

### Genome sequencing, assembly and general characeristics

We sequenced the genome of *P. chrysogenum* NCPC10086 using a whole-genome shotgun sequencing strategy [20, 21]. Owing to different sequencing technologies have various advantages and disadvantages [22, 23], we generated a high quality genome assembly using a combination of first and second generation sequencing platforms and strategies (Table 1). First, we generated single-end reads using Roche 454 pyrosequencing platform [24] and mate-pair reads with 3-4 Kb and 6-8 Kb insert fragment sizes, using ABI 3730 and MegaBACE 1000 Sanger sequencing platforms [25], respectively. Then we generated mate-pair reads with 1-2 Kb insert fragment size using Illumina HiSeq 2000 sequencing platform [26, 27] and used all mate-pair reads to join contigs into scaffolds. Overall, we get 204× sequencing coverage of high quality reads for *de novo* assembly (Table 1).

**Table 1:** *P. chrysogenum* NCPC10086 genome sequencing data

Instruments	Insert fragment size (Kb)	Reads length (bp)	Sequencing throughput (Mb)	Coverage
Roche 454 GS	single end	410	614	18×
Illumina HiSeq 2000	1-2	50	6,120	180×
ABI 3730	3-4	659	170	5×
MegaBACE 1000	6-8	739	34	1×
Total	-	-	6,938	204×

The estimated genome size of *P. chrysogenum* NCPC10086 is about 34 Mb.

We got a total genome size of 32.3 Mb (Table 2) similar as Wisconsin54-1255 [12]. The length of longest contig is 1,655 Kb, which indicates fine continuity of assembly. Owing to the deeper sequencing data, the contig N50 of NCPC10086 is 661 Kb which is 70% higher than Wisconsin54-1255 (389 Kb). The scaffold N50 of our genome is 2.8 Mb and the longest scaffold is 4,063 Kb, illustrating our genome is suitable for structural variations detection, especially large-scale translocations. The gene structures were predicted with a combined *de novo* and homology-based approach. Firstly, we masked all the repeat sequences in the genome and used Fgenesh (v 3.1.2)[28] and GeneMark-ES (v 2.3e)[29] to provide an initial set of 11,284 predicted ORFs. Secondly, we took advantage of the gene prediction results of Wisconsin54-1255 [12] to revise and complement our predicted genes by homology searches. At last, the former two results were integrated together, and 13,290 protein-coding genes were predicted in *P. chrysogenum* NCPC10086 genome (Table 2). GC content of the genome is 48.9% and every 2,430 bp has one gene. The mean gene length is 1,499 bp and most of the genes have introns.

**Table 2:** Global statistics of the genome assembly and annotation of *P. chrysogenum* NCPC10086

Assembly	Number	N50 (Kb)	Longest (Kb)	Size (Mb)	Percentage of the assembly
**Contigs**	327	661	1,655	32.2	99.7
**Scaffolds**	175	2,847	4,063	32.3	100
**Annotation**	**Number**	**Mean length**	**GC content (%)**	**Gene density** **(1 gene every n bp)**	
**Coding genes**	13,290	1,499	48.9	2,430	
**Genes with intron**	10,966	1,559	51.6	2,945	

**Figure 1:**
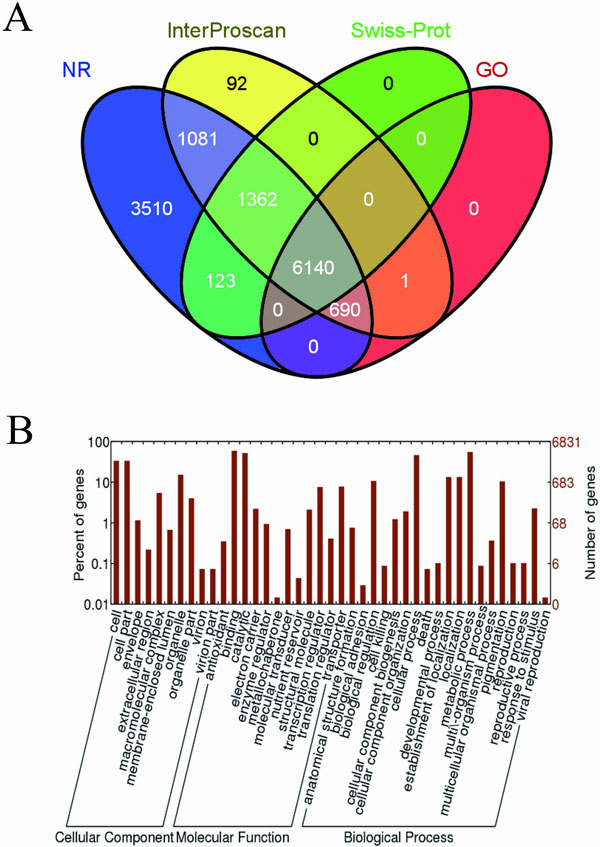
**Gene annotation and gene ontology of** ***P. chrysogenum*** **NCPC10086**. (A) Venn diagram showing unique and shared proteins could be annotated by databases of Non-redundant, InterProscan, Swiss-Prot/UniProtKB and Gene Ontology. (B) There are 6,831 proteins could be assigned to cellular component, biological process and molecular function by Gene Ontology classification system.

As to gene annotation, four databases of Non-redundant (NR), InterProscan [30, 31], Swiss-Prot/UniProtKB [32, 33] and Gene Ontology system (GO)[34, 35] were used to annotate 13,290 predicted genes of NCPC10086 (Figure 1A). We found 12,906 genes have homolog in NR, 9,371 genes have protein structural domains in InterProscan, and 7,625 genes have homolog in Swiss-Prot/UniProtKB, and 6,831 genes can be classified in GO. There are 6,140 genes can be annotated by all four gene annotation systems (Figure 1A), suggesting these genes are well studied. The second large genes group is NR-specific with the number of 3,510, which indicates functions of these genes are just beginning to be understood. The following two large groups are 1,362 genes with homolog in Swiss-Prot database and 1,081 genes with protein domain in InterProscan. Both of them cannot be assigned to GO system, indicating functions of these genes are little known but ready for deeper investigation in some extent. There are 6,831 genes can be assigned at least one GO term for describing cellular component, biological process and molecular function classification (Figure 1B).

### Genome comparison analysis between *P. chrysogenum* NCPC10086 and Wisconsin54-1255

**Figure 2:**
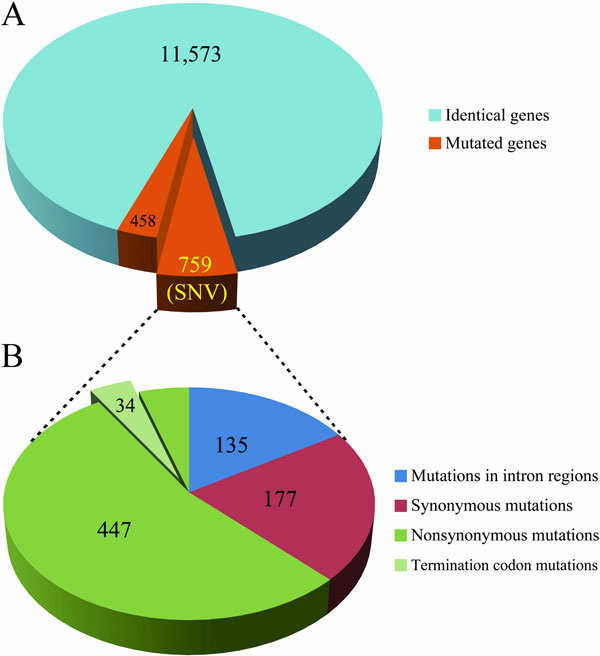
**The single nuclear variations (SNVs) statistics between NCPC10086 and Wisconsin54-1255**. (A) We discovered 11,573 genes are identical and 759 SNVs between two strains. (B) Among them, 135 SNVs take place in intron regions, 177 SNVs are synonymous mutations and 447 SNV are non-synonymous mutations including 34 termination codon mutations.

*P. chrysogenum* Wisconsin54-1255 is a low-penicillin producer strain widely used in libraries. The genome sequence of Wisconsin54 was well sequenced in 2008, which is the first published genome of *P. chrysogenum* [12]. The evolutionary relationship between Wisconsin54-1255 and our sequenced high-penicillin strain NCPC10086 is very close. Here we offer a comprehensive genome comparison analysis between these two *P. chrysogenum* strains, and try to figure out some interesting genomics discrepancy. To access comparative genomics statistics results, we aligned all the genes of Wisconsin54-1255 to the scaffolds of NCPC10086 to detect gene mutations. There are 12,943 predicted genes in Wisconsin54-1255 and 13,290 predicted genes in NCPC10086. And we discovered 11,573 genes are identical between two strains, 89% for Wisconsin54 and 87% for NCPC10086, which indicates these two genomes are very conservative (Figure 2A). As to the non-identical genes, 1,154 genes' identity are higher than 90%, part of them leading to mutations, and only 22 genes have less than 60% identity. In addition, we complemented 64 genes, which are partial in Wisconsin54-1255 and 514 noncoding sequence regions are redefined as protein-coding genes. Using Wisconsin54-1255 genome sequence as a reference, we realigned 50× high-quality short reads of NCPC10086 from Illumina HiSeq 2000 to identify single nuclear variations (SNVs). In order to differentiate sequencing errors from mutations, we used three thresholds to filter out unreliable mutation results: (1) we required at least five reads for each mutation; (2) average quality of each mutation had to be higher than 20; (3) there had to be at least one pair of mate-pair reads to support. We got 759 SNVs in coding regions and 1,813 SNVs in noncoding regions. There are 135 SNVs in intron regions, 177 are synonymous mutations and 447 are non-synonymous mutations, including 34 termination codon mutations. All SNVs result in coding region is described as Figure 2B. Furthermore, we found 35 deletions and 19 insertions in exon regions, 86 copy number variations (CNVs) with the total size of 176,684 bp. The polymorphic genes with non-synonymous mutations are listed in Additional file 1.

Besides, we found 69 new genes that do not exist in the available genome sequence of Wisconsin54-1255 [12]. We analyzed these new genes carefully and figured out several metabolism, biosynthesis and degradation (Table 3). Firstly, Pch125g10680, Pch106g00010, Pch114g00050 genes involved in amino sugar, nucleotide sugar metabolism, N-Glycan biosynthesis and oxidative phosphorylation may provide more energy in high-penicillin producer for penicillin synthesis. Secondly, we found another new gene, Pch106g00010, involved in nitrogen metabolism, which is up regulated strongly in cultures supplemented with the side chain precursor PAA (phenylacetic acid) in high-producing strain [36]. The last but not the least, the new gene, Pch018g00010, was discovered to participate in glutathione metabolism that may boost production of penicillin. The biosynthesis of cysteine is precursor for the penicillin biosynthesis, and the genes involved in this pathway were over expressed in the high-penicillin producing strain [12]. As to the previous study [37], the increase in the cysteine biosynthesis requires a large NADPH supply, but the oxidized glutathione under oxidative stress also requires NADPH, which could reduce the cysteine biosynthesis. So, we presume that glutathione metabolism may save NADPH and indirectly promote the penicillin production.

**Table 3:** Metabolism or progress involved by several "new" genes

Gene name	Length (bp)	Location (bp)	The metabolism or progress
Pch125g10680	4980	scaf125 (3,231,484-3,236,463)	Amino sugar and nucleotide sugar metabolism
Pch106g00010	769	scaf106 (193-961)	Nitrogen metabolism, oxidative phosphorylation
Pch114g00050	153	scaf114 (16,204-16,356)	Oxidative phosphorylation
Pch041g00010	713	scaf041 (112-824)	Riboflavin metabolism
Pch056g00010	787	scaf056 (36-822)	N-Glycan biosynthesis
Pch018g00010	694	scaf018 (325-1,018)	Glutathione, arachidonic acid, taurine and hypotaurine metabolism,
Pch180g00010	580	scaf180 (50-629)	Fluorobenzoate, chlorocyclohexane and chlorobenzene, toluene degradation

**Figure 3:**
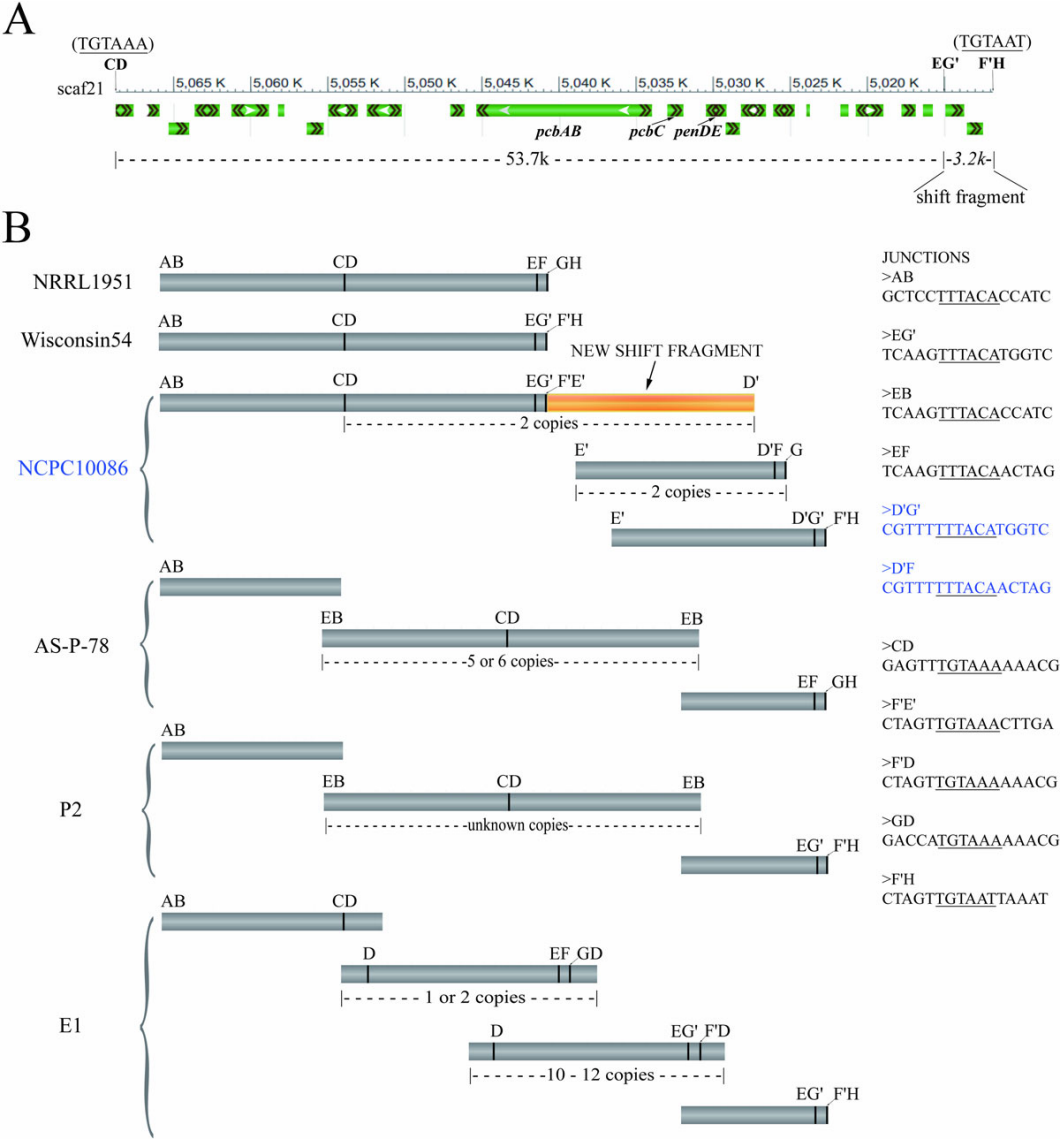
**Comparative organizations of penicillin biosynthetic genes cluster (PBC) in different strains**. (A) PBC region of Wisconsin54-1255 is about 56.9-kb, consisting of 53.7-kb fragment and 3.2-kb shift fragment bounded by a conserved TGTAAA/T hexanucleotide. (B) PBC fragment arrangement schematic. We discovered a new shift fragment in NCPC10086, marked with orange bar and blue letters.

To our best knowledge, the penicillin biosynthetic genes cluster (hereafter named PBC) located at chromosome I in *P. chrysogenum* is the dominant core for penicillin production [9, 38], which exist in all strains, including NRRL 1951 (wild-type) [39], Wisconsin54-1255, NCPC10086, P2 (Panlabs), AS-P-78 and E1 (Antibioticos, S.A.) [13]. Three penicillin biosynthetic genes, *pcbAB*, *pcbC* and *penDE* are gathered in this cluster (Figure 3A). Copy number and fragment arrangement are key features about PBC, which could impact on the yield of penicillin. PBC was amplified five to sixteen copies in different high-penicillin producers, such as five or six copies for AS-P-78 and twelve to fourteen copies for E1 [13] (Figure 3B). As to the low-productive strain Wisconsin54-1255, PBC just has one single copy with the length of 56.9 Kb, consisting of a 53.7 Kb fragment and a 3.2 Kb shift fragment bounded by a conserved TGTAAA/T hexanucleotide [8] (Figure 3A). Through reads coverage detection method, we found seven copies of PBC in NCPC10086 with the length of 56.9 Kb, including two copies for 110.6 Kb fragment of D--EG'--F'E'--D' and 56.9 Kb fragment of E'--D'F--G, and one copy for 56.9 Kb fragment of E'--D'G'--F' (Figure 3B). Compared with the previous investigations [8, 9, 13, 40], one PBC fragment arrangement in NCPC10086 is unique and has never been reported before, orange bar and blue letters shown in Figure 3B. It is a 53.7 Kb "NEW SHIFT FRAGMENT" in our genome. We believe the TTTACA hexanucleotide and its inverse complement TGTAAA could be hot spots for site-specific recombination after mutation with nitrosoguanidine. Unfortunately, the length of PBC is so long that we cannot get the full precise arrangement of these copies.

**Figure 4:**
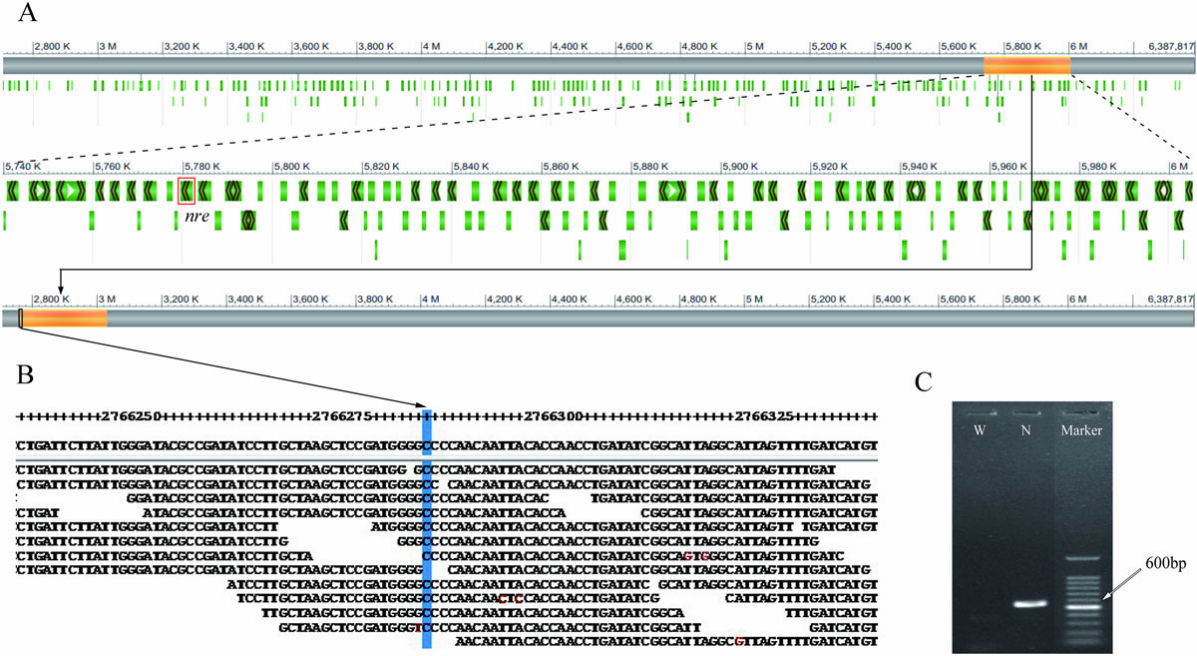
**Identification of 266 Kb translocation**. (A) A 266 Kb fragment translocation (orange bar) between Wisconsin54-1255 and NCPC10086. Genes are marked with green bar; special one is red boxed. (B) Reads alignment of the region around the breakpoint of translocation shows that there are 11 reads to support our conclusion. (C) PCR identification of the translocation. W stands for Wisconsin54-1255 and N stands for NCPC10086.

**Figure 5:**
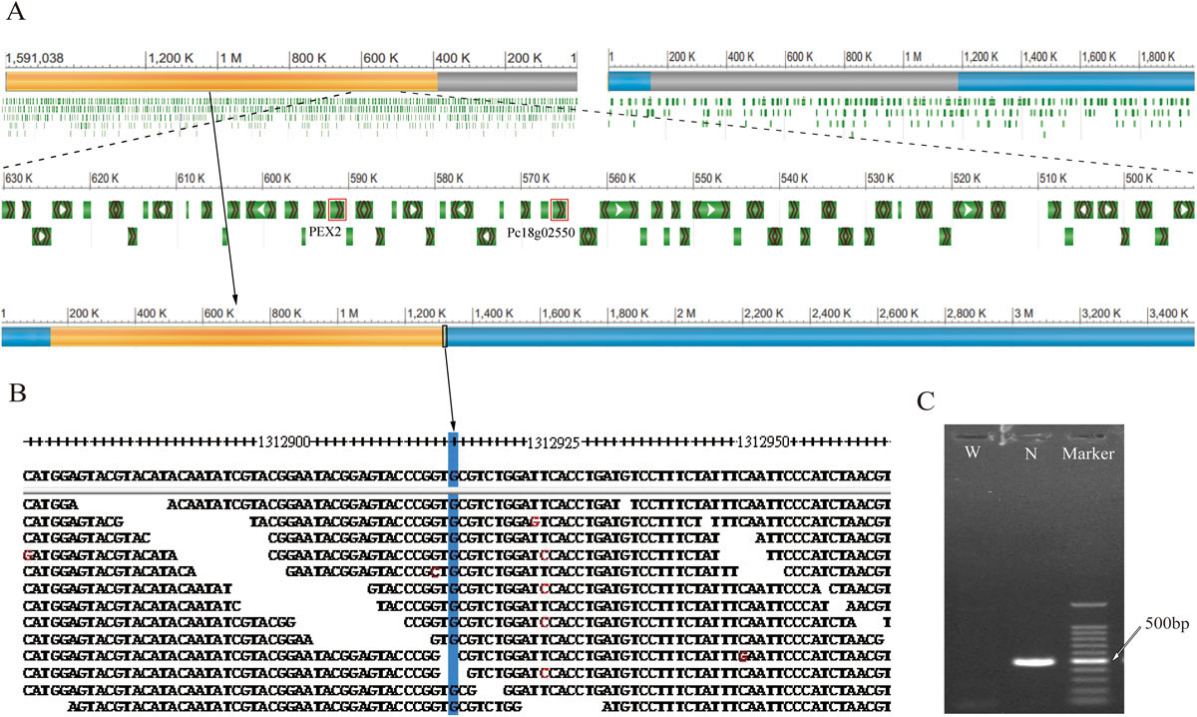
**Identification of 1,202 Kb translocation**. (A) A 1,202 Kb fragment translocation (orange bar) between Wisconsin54-1255 and NCPC10086. Genes are marked with green bar; special one is red boxed. (B) Reads alignment of the region around the breakpoint of translocation shows that there are 11 reads to support our conclusion. (C) PCR identification of the translocation. W stands for Wisconsin54-1255 and N stands for NCPC10086.

The genes involved in fungal secondary metabolic pathways share a common tendency towards physical cluster, with a preference for subtelomeric regions [41]. The wide range of translocation is a very interesting phenomenon happened in industrial filamentous fungus, for example, *Aspergillus niger* [16]. Comparison with Wisconsin54-1255, we found two large fragment translocations in NCPC10086. One is a 266 Kb fragment in subtelomere (position 5,739,429-6,005,263 in scaffold22 of Wisconsin54-1255) transferred to the around centromere in NCPC10086 and the range of translocation is about 3 Mb (Figure 4A). To avoid the possibility of assembly error, we realigned mate-pair reads around the breakpoint of translocation and found enough reads to across the breakpoint (Figure 4B). Moreover, we did PCR identification around the breakpoint and only NCPC (N) has band with estimated size (Figure 4C). This 266 Kb fragment of translocation includes 107 genes from Pc22g24360 to Pc22g25450 and their mean genes size is 1,638 bp. Unfortunately, the functions of most genes are unknown except for Pc22g24480 (*nre*), which encodes a regulator of nitrogen metabolite repression (red boxed in Figure 4A). We hypothesize that the translocation of Pc22g24480 may promote the nitrogen metabolism in NCPC10086, corresponding to the "new" gene discovered in nitrogen metabolism, which is described earlier in this article. Another translocation is a 1,202 Kb fragment (position 387,513-1,589,522 bp in scaffold18 of Wisconsin54-1255) transferred to scaffold072 of NCPC10086 starting at position 148,434 bp (Figure 5A). The 1,202 Kb fragment consists of 494 genes from Pc18g01610 to Pc18g06720 and their mean size is 1,480 bp. Those genes associate with energy metabolism and peroxisome pathway, such as Pc18g02420, which encodes mitochondrial ADP/ATP carrier, and Pc18g02550, which encodes PEX-2 (red boxed in Figure 5A). We also did mate-pair reads alignment around the breakpoint of translocation (Figure 5B) and PCR identification (Figure 5C) to certificate this translocation.

At last, we focused on some genes involved in homogentisate and the regulators of penicillin biosynthesis. Table 4 shows the comparison results of these genes between Wisconsin54-1255 and NCPC10086. *PahA* gene encodes a phenylacetate 2-hydroxylase that catalyzes the first step of phenylacetate catabolism, decreasing the precursor availability for penicillin biosynthesis [42]. Owing to a point mutation (C^598^→T) in *pahA* gene, the homogentisate pathway for PAA catabolism has been reported to be largely inactivated but the penicillin yield is increased in Wisconsin54-1255 [42]. Based on the comparative genomics analysis, we found *pahA* gene was shown in a 27.1 Kb translocation, but *pahA* gene is identical between the two strains. Three other genes, *pahB* (Pc22g02230), *pahC* (Pc16g01770) and *pahD* (Pc21g22560), are strongly similar to *pahA* and all of them belonging to cytochrome P450 monooxygenases. For *pahC* gene, there is a mutation (G^482^→ C) causes a single amino-acid substitution at position 129 of the protein: an alanine residue in strain Wisconsin 54-1255 has been substituted to proline in NCPC10086 strain (A129P). For *pahD* gene, we found a synonymous mutation (C^1976^→ T). All the promoter sequences of these genes are all the same. Pc*laeA* gene was found as a global regulator of secondary metabolism, including penicillin biosynthesis, sporulation and pigmentation in *P. chrysogenum* [43], which is identical in sequence in the genome of Wisconsin54-1255 and NCPC10086 with the same copy number. Due to Pc*laeA* gene encodes a velvet-like complex, we checked another velvet-like complex PcVelA. Its corresponding Pc*velA* gene is a key regulator of metabolism, acting both as an activator and repressor of secondary metabolism in *P. chrysogenum* [44]. There is a mutation ( C^1002^→ T) in Pc*velA* gene of NCPC10086 causes glutamine^315^ to stop codon (Q315Stop). Through functional domain scanning, we can see that position 28-238 is velvet factor non-mutated.

**Table 4:** Single nuclear variations (SNVs) involved in homogentisate pathway and the regulators of penicillin biosynthesis

Gene	Length (bp)	Description	Discrepancies (SNVs)
***pahA***	1,727	A phenylacetate 2-hydroxylase which catalyzes the first step of the homogentisate pathway for PAA catabolism	--
***pahB***	1,797	Strongly similar to *pahA*	--
***pahC***	1,785	Strongly similar to *pahA*	non-synonymous (G482C)
***pahD***	2,423	Strongly similar to *pahA*	synonymous (C1976T)
**Pc** ***laeA***	1,340	A global regulator of secondary metabolism	--
**Pc** ***velA***	1,745	An activator and repressor of secondary metabolism	non-synonymous (C1002T)

## Discussion

We sequenced the whole genome of an industal high-penicillin producing strain NCPC10086 and provided an integral whole geome comparison results with Wisconsin54-1255. A total genome size of 32.3 Mb was assembled with contig N50 of 661 Kb and scaffold N50 of 2.8 Mb. The gene structures were predicted with a combined *de novo* and homology-based approach, and annotated by four gene annotation systems.

By whole genome comparative analysis, we observed a large number of mutations, insertions and deletions, and structural variations. There are 69 "new" genes that not exist in the genome sequence of Wisconsin54-1255 and some of them are involved in energy metabolism, nitrogen metabolism and glutathione metabolism. As was expected, the high-penicillin producing strain needs more energy for penicillin synthesis, sorting, transport and processing, and we confirm some new genes participate in it. One "new" gene was discovered in nitrogen metabolism, which is up regulated strongly in cultures supplemented with the side chain precursor PAA (phenylacetic acid) in high-producing strain [36]. Both cysteine biosynthesis and the oxidized glutathione need NADPH, if glutathione metabolism is more active, NADPH could be reserved for more cysteine biosynthesis to improve the penicillin production. Our "new" gene involved in glutathione metabolism may impact on this process.

The penicillin biosynthetic genes cluster (PBC) is the well-known dominant core for penicillin production existing in all strains; copy number and fragment arrangement are the key features for PBC. The high-penicillin producing strain, NCPC10086, has seven copies of PBC and one 53.7 Kb "new shift fragment" with unique arrangement type. The TTTACA sequence and its inverse complement TGTAAA sequence could be hotspots for site-specific recombination after multiple mutations. This process may aim to repair damage from mutations by nitrosoguanidine. We found two large translocations in NCPC10086; one is a 266 Kb fragment in subtelomere transferred to centromere including genes regulating nitrogen metabolite repression; another is a 1,202 Kb fragment consists of a mitochondrial ADP/ATP carrier involved in energy metabolism and peroxin-2 gene involved in peroxysome pathway.

Due to our comparative genomics statistics results, we predicted that energy metabolism and nitrogen metabolism plays an important role in penicillin production together with glutathione metabolism and peroxysome pathway. To further analysis genes involved in those processes, we looked into two types of genes deeper, *pahA* gene set and velvet-like complex genes. Translocation, stop codon mutation, synonymous and non-synonymous mutations are found there. These variations may impact the homogentisate pathway for PAA catabolism as well as global regulation of secondary metabolism, including penicillin biosynthesis, sporulation and pigmentation.

We found out many mutations and structural variations, but how many of them and how they affect the penicillin yield is still a formidable challenge. Efficient approaches to narrow down the possibilities are to sequence more genomes for common variations and system biological investigation using "omic" data [45]. Through genome resequencing and functional analysis, identification of precise mutations in strains with altered phenotypes will add insight into specific gene functions and guide further metabolic engineering efforts.

## Conclusions

This is the first high-quality genome of high-penicillin producing industrial stain of *Penicilliumchrysogenum*, which can provides abundant genetic information for broad biomedical researchers. Through comparative genomics analysis with low-producing strain, we found a lot of mutations, insertions and deletions, and structural variations. Moreover, we showed some "new" genes not existent in the public genome sequence of Wisconsin54-1255 involved in energy metabolism, nitrogen metabolism and glutathione metabolism. Most remarkably, for the penicillin biosynthesis cluster, we are surprised to find a 53.7 Kb new "shift fragment" in our high-producing strain and the type of fragment arrangement is unique. In addition, we addressed a 266 Kb translocation including a regulator of nitrogen metabolite repression and a 1,202 Kb translocation including genes involved in energy metabolism and peroxysome pathway. Our findings lay a foundation for the insights into the high-penicillin producing mechanism and metabolic engineering in the future.

## Methods

### Source of sample and culture conditions

*P. chrysogenum* NCPC10086 strain was selected for genome sequencing as it was commercialized in North China Pharmaceutical Group Corporation. Spore suspensions of NCPC10086 were inoculated in 40 mL of seed medium (20 g/L sucrose corn steep liquor, 20 g/L sucrose, 5 g/L yeast extract, 5 g/L CaCO_3_, pH 5.8) in 250 ml flasks and incubated on a rotary shaker (250 r.p.m.) at 26°C for 24 h. Two milliliters of seed culture were transferred to 40 mL of fermentation medium (35 g/L lactose, 30 g/L corn steep liquor, 5 g/L (NH4)_2_SO4, 1 g/L KH_2_PO_4_,1 g/L, K_2_SO_4_, 10 g/L CaCO_3_, 2 g/L phenylacetic acid, 6 ml/L corn oil, pH 6.0) and grown at 26°C with shaking at 250 r.p.m.

### Genome sequencing and assembly

The genome of *P. chrysogenum* NCPC10086 strain was sequenced by whole genome random sequencing method [20, 21]. We used Roche 454 GS FLX system to produce 18× coverage single-end reads with an average read length of 410 bp to do contig assembly. Moreover, 3-4 Kb and 6-8 Kb mate-pair libraries were produced to do contig and scaffold assembly, with 5× coverage sequenced by ABI 3730 system and 1× coverage sequenced by Megabace1000 system. ABI 3730 and Megabace1000 produced an average read length of 659 bp and 739 bp respectively. Phred and Phrap [46, 47] were used to deal with the raw data from ABI 3730 and Megabace1000. Hybrid assembly was performed using Newbler [24] with all single-end and mate-pair reads by overlap-layout-consensus strategy. After assembly, we aligned our contigs to the reference sequence [12] to predict the gaps sizes distribution in our genome. According to the gaps size distribution, we designed 1-2 Kb mate-pair inserted fragment library to do scaffolding. We mapped the mate-pair high quality reads onto the scaffolds and used the reads to fill the gaps if one of the mate-pair reads located at the edge of gaps. At last, redundant sequences were deleted through self-alignment.

### Gene prediction and annotation

The repeat sequences of *P. chrysogenum* NCPC10086 were masked throughout the genome using RepeatMasker (v 3.3.0) and the RepBase library (20120418) [48] with Tandem Repeats Finder (v 3.2.1)[49]. The gene structures were predicted with a combined *de novo* and homology-based approach. Firstly, for all repeat-masked scaffolds larger than 1 Kb, Fgenesh (v 3.1.2)[28] and GeneMark-ES (v 2.3e)[29] were performed on the whole genomic sequence to provide an initial set of predicted ORFs. Fgenesh is trained on sequences of *Penicillium funiculosum* and GeneMark-ES is upon the self-training algorithm for fungal genomes. Preference was given to Fgenesh genes, and all predicted protein should be larger than 10aa. Secondly, we use the gene prediction results of *P. chrysogenum* Wisconsin54-1255 to revise and complement our predicted genes by homology searching. At last, the former two results were integrated together as predicted genes. All predicted proteins were blastp [50] against databases of GenBank's non-redundant proteins, InterProscan [30], Swiss-Prot/UniProtKB [32] and Gene Ontology (GO), and the best alignment of every protein was considered its annotation. No alignment results by blastp of predicted proteins were automatically considered as hypothetical proteins. We presented unique and shared proteins among four gene annotation systems by venn diagram (http://bioinfogp.cnb.csic.es/tools/venny/index.html). WEGO [51] was used to plot GO annotation results. The pathway analysis is carried out by KAAS (v 1.67x) with SBH method [52].

### Calling single nuclear variations (SNVs), insertions and deletions (InDels) and copy number variation (CNVs)

Based on the assembled *P. chrysogenum* Wisconsin54-1255, we realigned all of the high-quality reads with the genome by SOAP(v 2.21) [53] to identify the SNVs. In the reads gap-free alignment process, at most two mismatches were allowed between a read and the reference, and best hits were selected. Multiple reads mapping results were filtered. We use SOAPsnp (v1.03)[54], a statistical model based on Bayesian theory and Illumina quality system, to calculate a probability for each possible genotype at each position on the reference genome. We used five thresholds to filter out unreliable SNV results: (1) we required at least five reads for each SNV; (2) average quality of each SNV had to be higher than 20; (3) the overall depth had to be less than 150; (4) the approximate copy number of flanking sequences had to be less than 2 (to avoid misreading SNV caused by the alignment of similar reads from repeat units or by copy number variations); (5) there had to be at least one pair of mate-pair reads to support. For InDel detection, we use Pindel(v0.2.4) [55] to find breakpoints of large deletions and medium-sized insertions from paired-end or single reads. The short reads alignment is by BWA- backtrack (v0.5.9) [56] and long reads is by BWA-SW [57]. We use SAM Tools(v0.1.17) [58] to manipulate alignments in the SAM format. Copy number variations detection is by CNV-seq [59] which is based on a robust statistical model with 50× high-quality reads from Illumina HiSeq 2000.

### Identification of structural variations

We used Blat (v 34) [60] with default parameter to align scaffolds of *P. chrysogenum* NCPC10086 to the reference, whole genome of *P. chrysogenum* Wisconsin54-1255, to search colinearity between them. The alignment results of each scaffold indicate a candidate location of the scaffold. For scaffolds with multiple hits, the top ten hits with highest sequence similarity remained as candidate locations. The alignment with the longest matches in a linear orientation between a scaffold and the reference was picked as 'best-hit' of the scaffold. After finding structural variations, we use Blastn with parameter '1e^-5^' to check the detail alignment results. We randomly pick up 20× mate-pair reads from 180× high-quality Illumina HiSeq 2000 reads. Reads mapping is by SOAP (v 2.21) [53] and the alignment result is visualized by MapView (v 3.4.1) [61]. The 5'-3' primers of PCR identification of structural variations of "266 Kb translocation" are ACCTGGCGTGCCTCATGCAGCG and TTGGGGTGGAATGACGTGGGG, which are before 200 bp and after 300 bp of the breakpoint. The 5'-3' primers of PCR identification of structural variations of "1,202 Kb translocation" are ACCTGTGGGGATCATTAGCCTCC and ACTCGGATAGTCTAGGTTCGGCGG, which are before 250 bp and after 220 bp of the breakpoint.

## Availability of supporting data

*P. chrysogenum* strain NCPC10086 genome sequences are available via GenBank/EMBL/DDBJ under the accession APKG00000000.

## Electronic supplementary material

Additional file 1: **The list of polymorphic genes with non-synonymous mutations**. (XLS 134 KB)
